# Empowering Black Caregivers with Culturally Tailored Blood Pressure Self-Management Education

**DOI:** 10.1093/geroni/igaf122.4382

**Published:** 2025-12-31

**Authors:** Fayron Epps, Shanae Rhodes, Derrick Ndukwe, Leonarda Daudi, Solee Wirba, Ellen Mikus

**Affiliations:** UT Health San Antonio, San Antonio, Texas, United States; The University of Texas Health Science Center at San Antonio, San Antonio, Texas, United States; UT Health San Antonio, University of Texas Health Science Center San Antonio, Texas, United States; UT Health San Antonio, University of Texas Health Science Center San Antonio, Texas, United States; UT Health San Antonio, University of Texas Health Science Center San Antonio, Texas, United States; UT Health San Antonio, University of Texas Health Science Center San Antonio, Texas, United States

## Abstract

Hypertension is highly prevalent among Black caregivers, reflecting broader trends in the Black community, where nearly 56% of Black adults have hypertension. Black caregivers, who often experience chronic stress, financial strain, and limited access to healthcare, face an even greater risk. Despite these risks, many Black caregivers do not regularly monitor their blood pressure, highlighting the need for targeted interventions and self-management resources to reduce health risks in this vulnerable population. Therefore, this project aimed to enhance hypertension awareness among the Black community, especially caregivers. We evaluated the feasibility and acceptability of delivering culturally tailored blood pressure self-management education to Black caregivers at the 2025 Alter Dementia Summit. After the session, attendees completed pledge cards outlining their commitments and plans toward blood pressure self-management, with 118 responses collected. A content analysis identified eight key categories that reflect the ways in which the education influenced attendees’ intentions for blood pressure management: regularly monitoring blood pressure, engaging in physical activities, gaining and sharing knowledge about blood pressure management, improving nutritional practices, prioritizing overall health, enhancing medication management, reducing stress, and achieving proper rest and sleep. Overall, the pledges reflect a meaningful shift toward proactive, empowered, and holistic health behaviors inspired by the education. Educating and providing resources to populations disproportionately affected by cardiovascular and dementia-related disorders is crucial for disease prevention. This feasibility and acceptability study serves as a foundational step in developing an educational packet that can be integrated into existing forums to promote healthy blood pressure and dementia risk reduction.

